# One-step synthesis of PbSe-ZnSe composite thin film

**DOI:** 10.1186/1556-276X-6-324

**Published:** 2011-04-12

**Authors:** Seishi Abe

**Affiliations:** 1 Research Institute for Electric and Magnetic Materials, Sendai 982-0807, Japan

## Abstract

This study investigates the preparation of PbSe-ZnSe composite thin films by simultaneous hot-wall deposition (HWD) from multiple resources. The XRD result reveals that the solubility limit of Pb in ZnSe is quite narrow, less than 1 mol%, with obvious phase-separation in the composite thin films. A nanoscale elemental mapping of the film containing 5 mol% PbSe indicates that isolated PbSe nanocrystals are dispersed in the ZnSe matrix. The optical absorption edge of the composite thin films shifts toward the low-photon-energy region as the PbSe content increases. The use of a phase-separating PbSe-ZnSe system and HWD techniques enables simple production of the composite package.

## Introduction

Quantum-dot solar cells have attracted much attention because of their potential to increase conversion efficiency [1]. Specifically, the optical-absorption edge of a semiconductor nanocrystal is often shifted due to the quantum-size effect. The optical band gap can then be tuned to the effective energy region for absorbing maximum intensity over the solar radiation spectrum. Furthermore, quantum dots produce multiple electron-hole pairs per photon through impact ionization, whereas bulk semiconductors produce one electron-hole pair per photon.

Wide-gap semiconductor sensitized by quantum dot is a candidate material for such use. The quantum dot supports absorbing visible and near-infrared light. Up to now, various nanocrystalline materials (InP [2], CdSe [3], CdS [4,5], PbS [6], and Ge [7]) have been investigated as the sensitizer for TiO_2_. Alternatively, a wide-gap semiconductor ZnO is also investigated, since the band gap and the energetic position of the valence band maximum and conduction band minimum of ZnO are very close to that of TiO_2 _[8]. Most of these composite materials were synthesized through chemical techniques, however, physical deposition, such as sputtering, is also useful. In the material design for co-sputtering, based on the heat of formation, nanocrystal and matrix are clearly phase-separated in spite of the co-deposition from multiple sources [9,10]. However, it is generally found that sputtering techniques often damage a film due to contamination of the fed gas and high-energy bombardment of the film surface. Thermal evaporation in a high-vacuum atmosphere seems to be better as a preparation technique from the point of view of film quality. In addition, the present study focuses on the insolubility of the material system, since simultaneous evaporation from multiple sources often provides a solid solution [11]. The PbSe-ZnSe system is a candidate for the composite. In the bulk thermal equilibrium state, the mutual solubility range is quite narrow, less than 1 mol%, at temperatures below 1283 K [12]. In addition, a composite thin film of PbSe nanocrystal embedded in ZnSe matrix is capable of exhibiting the quantum size effect because of the relatively large exciton Bohr radius of 46 nm in PbSe [13] and the relatively wide band gap of 2.67 eV in ZnSe [14]. Hence, the optical gap of PbSe nanocrystals will probably be tuned to the maximum solar radiation spectrum. The dendritic PbSe nanostructure [15] and ZnSe nanobelt array [16], for instance, are hitherto investigated, but there is no report for one-step synthesis of PbSe-ZnSe composite thin film. Furthermore, an evaporation technique should be carefully selected, since the techniques involving a thermal non-equilibrium state, such as molecular beam epitaxy, increase the solubility limit [17]. The use of hot-wall deposition (HWD), which can provide an atmosphere near thermal equilibrium, is therefore indicated here [18]. Based on these considerations, one-step synthesis of a PbSe-ZnSe composite thin film was investigated by simultaneous HWD from multiple sources for the first time.

## Experimental details

**Figure 1:**
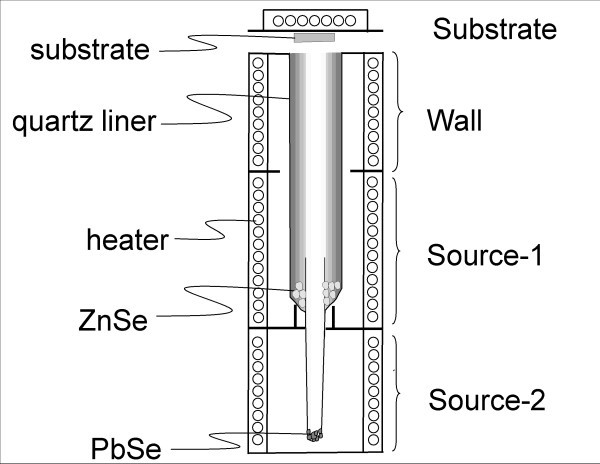
**HWD apparatus used in the study**. It consists of four electric furnaces for substrate, wall, source-1, and source-2.

A PbSe-ZnSe composite thin film was prepared by the HWD method. Figure 1 is a schematic diagram of the HWD apparatus used. There were four electric furnaces in the apparatus, designated as substrate, wall, source-1, and source-2. Each temperature could be controlled independently. In the HWD method, deposition and re-evaporation are continuously repeated upon a film surface, resulting in achieving a state near thermal equilibrium [18]. PbSe and ZnSe were used as evaporation sources and were synthesized from elements of Pb, Zn, and Se with 6 N purity. The PbSe and ZnSe sources were located at furnaces of source-2 and source-1 for simultaneous evaporation to a glass substrate (Corning #7059). Here, the temperatures were kept constant at 573 K for the substrate, 773 K for the wall, and 973 K for source-1 (ZnSe). The source-2 (PbSe) temperature was varied from 763 to 833 K to provide different PbSe concentrations. The PbSe-ZnSe composite thin film was structurally characterized using X-ray diffraction (XRD) with Cu Kα radiation (Rigaku RAD-X). A symmetric θ-2θ configuration was used. The optical absorption spectrum of the film was observed using a UV-vis-IR spectrometer (Shimadzu UV5300). The composition of the film was analyzed using energy dispersive spectroscopy (EDX model: Phoenix). The film was directly observed by transmission electron microscopy (TEM) operating at 300 kV (Hitachi H-9000NAR). In the sample preparation, mechanical polishing, dimpling, and ion milling were performed. Nanoscale elemental mapping was performed using scanning transmission electron microscopy (STEM, Hitachi HD-2700) in EDX mode (EDAX model: Genesis) operating at 200 kV with an energy resolution of approximately 145 eV.

## Results and discussion

**Figure 2:**
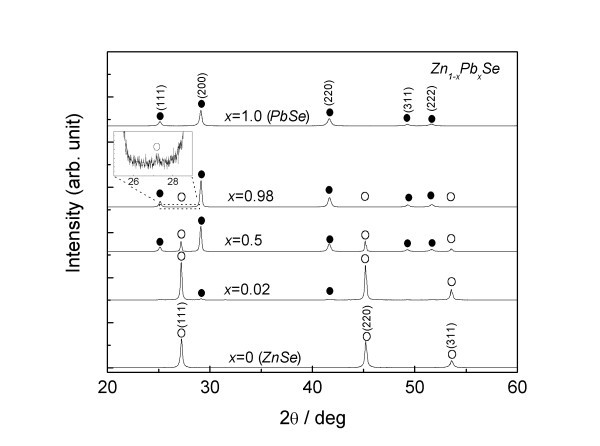
**XRD pattern of powder-synthesized Zn_1-*x*_Pb*_x_*Se with respect to *x***. Dots indicate PbSe and circles indicate ZnSe.

The bulk PbSe-ZnSe phase diagram is now revealed at ZnSe concentrations below 45 at.% (Pb-rich side) [12], although the phase diagram of the Zn-rich side still remains unclear. Powder synthesis of a PbSe-ZnSe system was investigated prior to investigating the film preparation. Figure 2 depicts the powder XRD pattern of the Zn_1-*x*_Pb*_x_*Se system. In the powder synthesis, the bulk PbSe and ZnSe thus synthesized was used as starting materials. The desired composition of the system was prepared in an agate mortar and vacuum-sealed in a quartz tube for heat treatment at 1273 K for 48 h. Finally, the samples were successively water-quenched to maintain the solubility range at a synthesis temperature then crushed into powder for the following experiment setup. At *x *= 0, all of the XRD peaks are assigned to the zinc-blend structure of ZnSe, with a lattice constant of 0.5669 nm, estimated from the XRD peaks in a high-2θ range from 100° to 155°, using the Nelson-Riley function [19]. The XRD peak of PbSe with an NaCl structure appears at Pb concentrations exceeding 0.02. The lattice constant of the ZnSe at *x *= 0.02 is the same as at *x *= 0, within the precision of the experiment technique. This result indicates that the solubility range of Pb in ZnSe is negligible. In contrast, the lattice constant of PbSe is estimated to be 0.6121 nm at *x *= 1.0 and 0.6117 nm at *x *= 0.98. A slight decrease in the lattice constant is seen in PbSe, due to the difference in ionic radii of Pb and Zn. Weak XRD peaks of ZnSe are also observed at *x *= 0.98 as seen in the inset for easier viewing. This result indicates that the solubility range of Zn in PbSe is less than 0.02 at 1273 K. The result is in good agreement with the previous result [12]. The phase separation of the PbSe-ZnSe system is thus also seen on the Zn-rich side in the thermal-equilibrium state. The film preparation for PbSe-ZnSe composite is next investigated based on these results.

The two sources were simultaneously evaporated to prepare a PbSe-ZnSe composite thin film. In the apparatus used, thermal radiation from the wall- and the source-furnace induced an unintentional increase of the substrate temperature up to 515 K without use of the substrate-furnace. The deposition rate of the film was almost the same irrespective of the substrate temperature in the range from 515 to 593 K. A homogeneous color is observed visually in these films. Above a substrate temperature of 593 K, the deposition rate abruptly decreased with increasing temperature, since re-evaporation of PbSe from the film surface became dominant. The films visually exhibit an inhomogeneous yellowish and metallic color, probably caused by a significant reduction in the PbSe while the ZnSe remained, due to the relatively high vapor pressure of PbSe [20]. The wall temperature also induced similar behavior. A substrate temperature of 573 K and a wall temperature of 773 K are therefore adopted throughout the present study.

**Figure 3:**
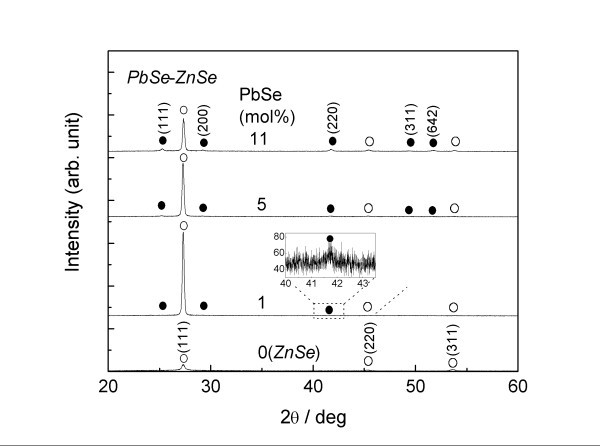
**XRD pattern of the PbSe-ZnSe composite thin films**. Dots indicate PbSe and circles indicate ZnSe.

Figure 3 depicts the XRD pattern for the PbSe-ZnSe composite thin films. The weak XRD peak of PbSe at 1 mol% is enlarged in the inset for easier viewing. At a PbSe concentration of 0 mol% (i.e., pure ZnSe), polycrystalline ZnSe with a zinc-blend structure is observed, with PbSe phase appearing at concentrations exceeding 1 mol%. The solubility range of Pb in ZnSe is therefore found to be quite narrow, less than 1 mol%, corresponding well to the bulk result (Figure 2). The composite films thus deposited on a glass substrate exhibit a reasonably polycrystalline structure, but dominant (111) growth is seen in the ZnSe phase irrespective of *x*. At 1 mol%, the lattice constant at the PbSe (220) peak is estimated to be 0.6118 nm, close to that of the bulk result (Figure 2). This result suggests that there is also a narrow solubility range on the Pb-rich side. The phase-separating PbSe-ZnSe system is therefore maintained not only in the bulk product, but also in the film thus obtained, despite the simultaneous evaporation from multiple sources. This result demonstrates that an atmosphere near thermal equilibrium was achieved in the HWD apparatus used.

**Figure 4:**
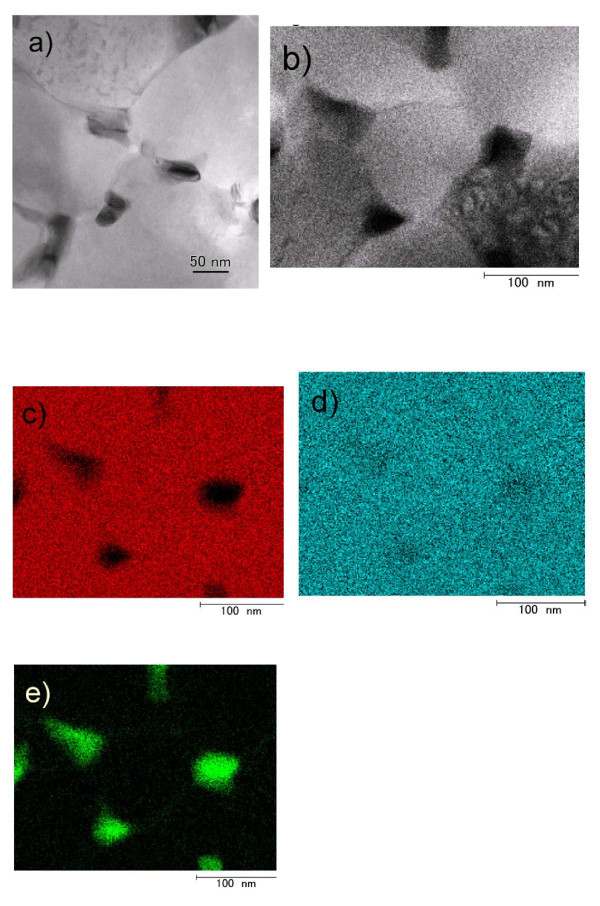
**Direct observation of PbSe-ZnSe composite thin film containing 5 mol% PbSe**. **(a) **Bright-field TEM image. **(b) **Bright-field image of STEM mode. **(c) **Elemental mapping of Zn (red), **(d) **Se (blue), and **(e) **Pb (green).

Figure 4a presents a bright-field TEM image of the PbSe-ZnSe composite thin film containing 5 mol% PbSe. Dark isolated grains with sizes of 25 to 50 nm are seen dispersed along the grain boundary of the bright area. Figure 4b-e presents an STEM-EDX elemental mapping of the sample through X-ray detection of Zn K (red), Se K (blue), and Pb L (green). Similar morphology is also seen in the bright-field STEM image (Figure 4b)). The dark grains indicate the absence of elemental Zn (Figure 4c) and the presence of Se and Pb (Figure 4d, e). It is thus determined that the dark grains are nanocrystalline PbSe. The other region is widely covered with the elements Zn and Se (Figure 4c,d), reasonably assumed to compose ZnSe. It is therefore determined that isolated PbSe nanocrystals are dispersed in the ZnSe matrix. The nanocrystals are estimated to be sufficiently small to exhibit the quantum-size effect because of the exciton Bohr radius of 46 nm in PbSe [13].

**Figure 5:**
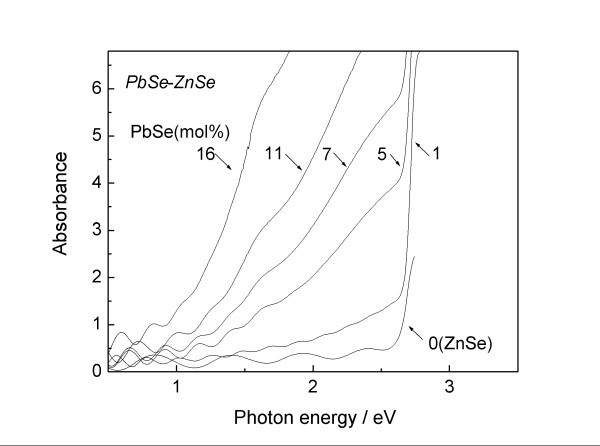
**Optical absorption spectra for PbSe-ZnSe composite thin films**.

Figure 5 depicts optical absorption spectra for the PbSe-ZnSe composite thin films. For comparison, the spectrum of a pure ZnSe thin film is also presented in the figure. PbSe and ZnSe have direct band structures [21,22], and an intact absorbance is employed here to exactly evaluate the absorption edge. At a 0 mol% PbSe, the optical absorption edge of ZnSe is clearly observed at 2.7 eV. Weak absorption then broadly appears at a PbSe concentration of 1 mol% in the visible region, together with the optical absorption edge of ZnSe. Such multiple absorptions are also seen in the spectra at concentrations up to 7 mol%, indicating the obvious phase separation of the PbSe-ZnSe system. The broad absorption edge shifts toward the lower-energy region as the PbSe content increases. In particular, onset absorption can be confirmed at approximately 1.0 eV at 16 mol% PbSe, favorably covering the desirable energy region for high conversion efficiency [23]. Therefore, it should be noted that the PbSe-ZnSe composite thin film exhibits the valuable characteristic of vis-NIR absorption. However, it is unclear whether the shift of the optical absorption edge is due to the PbSe nanocrystals, since the mean grain size of the PbSe remains almost the same at 27 nm irrespective of the PbSe content, according to the XRD result (Figure 3) using Scherrer's equation [24]. The minimal appearance of infrared absorption at 16 mol% PbSe strongly suggests that relatively large-scale PbSe grains are partially involved in the composite film, since the energy band gap of bulk PbSe is 0.27 eV [22]. Another TEM image also indicates the presence of relatively large PbSe crystals of approximately 100 nm, even with a small amount of 5 mol% PbSe (not shown here). Hence, the mean grain size of the PbSe is bimodally distributed in the composite. These large-scale PbSe grains probably dominate the full width at half maximum value of the XRD peak, resulting in no obvious relation between the optical absorption shift and the PbSe grain size. The size control of the nanocrystalline PbSe is therefore insufficient in the present study. The substrate temperature thus adopted seems to assist in the aggregation of PbSe nanocrystals. However, a one-step synthesis of the composite package has the potential to lead to low-cost production of next-generation solar cells.

## Conclusion

We investigated the preparation of PbSe-ZnSe composite thin films by a co-evaporating HWD method. The relatively high substrate and wall temperatures induce re-evaporation of PbSe from the substrate surface while the ZnSe remains. The solubility limit of Pb in ZnSe is quite narrow, less than 1 mol% in the film form, indicating that an atmosphere near thermal equilibrium is achieved in the apparatus used. Elemental mapping indicates that isolated PbSe nanocrystals are dispersed in the ZnSe matrix. The optical absorption edge shifts toward the lower-photon-energy region as the PbSe content increases. In particular, onset absorption can be confirmed at approximately 1.0 eV with 16 mol% PbSe, favorably covering the desirable energy region for high conversion efficiency. The insolubility material system and the HWD technique enable a one-step synthesis of PbSe-ZnSe composite thin film. Further investigation is needed to produce a narrower size distribution of the PbSe nanocrystals through the use of a single-crystal substrate, for instance, to control the growth direction, or through using a different phase-separating material system.

## Competing interests

The authors declare that they have no competing interests.
